# Regulated Tristetraprolin Overexpression Dampens the Development and Pathogenesis of Experimental Autoimmune Uveitis

**DOI:** 10.3389/fimmu.2020.583510

**Published:** 2021-01-25

**Authors:** Biying Xu, Jihong Tang, Cancan Lyu, Wambui S. Wandu, Deborah J. Stumpo, Mary J. Mattapallil, Reiko Horai, Igal Gery, Perry J. Blackshear, Rachel R. Caspi

**Affiliations:** ^1^ Laboratory of Immunology, National Eye Institute, NIH, Bethesda, MD, United States; ^2^ Signal Transduction Laboratory, National Institute of Environmental Health Sciences, Research Triangle Park, NC, United States; ^3^ Departments of Medicine and Biochemistry, Duke University Medical Center, Durham, NC, United States

**Keywords:** experimental autoimmune uveitis (EAU), autoimmunity, inflammatory cytokine, IL-17, IFNγ, immunoregulation, tristetraprolin (TTP)

## Abstract

Non-infectious uveitis, a common cause of blindness in man, is often mediated by autoimmunity, a process in which cytokines play major roles. The biosynthesis and secretion of pro-inflammatory cytokines are regulated in part by tristetraprolin (TTP), an endogenous anti-inflammatory protein that acts by binding directly to specific sequence motifs in the 3’-untranslated regions of target mRNAs, promoting their turnover, and inhibiting synthesis of their encoded proteins. We recently developed a TTP-overexpressing mouse (TTPΔARE) by deleting an AU-rich element (ARE) instability motif from the TTP mRNA, resulting in increased accumulation of TTP mRNA and protein throughout the animal. Here, we show that homozygous TTPΔARE mice are resistant to the induction of experimental autoimmune uveitis (EAU) induced by interphotoreceptor retinoid-binding protein (IRBP), an established model for human autoimmune (noninfectious) uveitis. Lymphocytes from TTPΔARE mice produced lower levels of the pro-inflammatory cytokines IFN-γ, IL-17, IL-6, and TNFα than wild type (WT) mice. TTPΔARE mice also produced lower titers of antibodies against the uveitogenic protein. In contrast, TTPΔARE mice produced higher levels of the anti-inflammatory cytokine IL-10, and had higher frequencies of regulatory T-cells, which, moreover, displayed a moderately higher per-cell regulatory ability. Heterozygous mice developed EAU and associated immunological responses at levels intermediate between homozygous TTPΔARE mice and WT controls. TTPΔARE mice were able, however, to develop EAU following adoptive transfer of activated WT T-cells specific to IRBP peptide 651–670, and naïve T-cells from TTPΔARE mice could be activated by antibodies to CD3/CD28. Importantly, TTPΔARE antigen presenting cells were significantly less efficient compared to WT in priming naïve T cells, suggesting that this feature plays a major role in the dampened immune responses of the TTPΔARE mice. Our observations demonstrate that elevated systemic levels of TTP can inhibit the pathogenic processes involved in EAU, and suggest the possible use of TTP-based treatments in humans with uveitis and other autoimmune conditions.

## Introduction

Non-infectious uveitic conditions have been estimated to cause 10%–15% of cases of blindness in western countries, and can be manifested as localized eye inflammation, or as part of a systemic syndrome (1, 2). The term “uveitis” is used to define a family of eye conditions with intraocular inflammation, and includes diseases such as sympathetic ophthalmia, “birdshot” chorioretinopathy, Behcet’s disease, sarcoidosis, and Vogt-Koyanagi-Harada disease (3, 4).

Cellular autoimmune processes are assumed to play major roles in the pathogenesis of these diseases, an assumption supported by the similarity between the pathological changes specific to these diseases and the typical ocular changes seen in rodents with experimental autoimmune uveitis (EAU), an autoimmune disease model mediated by immunopathogenic T-cells (3, 5). In mice, EAU is induced by immunization with the retinal antigen, interphotoreceptor retinoid-binding protein (IRBP) (6), or by adoptive transfer of T-cells sensitized against this retinal protein (7, 8).

Compounds such as glucocorticoids or cyclosporin have been the mainstays of treatment for non-infectious uveitis (9). More recently, “biologic” therapies targeting TNFα have been used, based on the apparent involvement of TNFα in the pathogenesis of EAU (10), as well as evidence that TNFα levels are increased in aqueous humor of patients with uveitis (11).

Tristetraprolin (TTP) is an inducible endogenous anti-inflammatory protein that binds to AU-rich sequences in its target mRNAs and promotes their deadenylation and subsequent decay (reviewed in (12)). This results in decreased expression of the proteins encoded by the mRNA targets, and provides a mechanism for the post-transcriptional regulation of expression of proteins whose transcripts contain appropriate AU-rich binding sites. Mice totally deficient in TTP develop a severe, systemic inflammatory syndrome, whose most prominent features include widespread destructive arthritis, cachexia, myeloid hyperplasia, conjunctivitis, dermatitis, and osteopenia (13). Many of these features are prevented by treating newborn TTP KO mice with antibodies to TNFα, or simultaneously knocking out both TNFα receptors, leading to the identification of TNFα mRNA as the first TTP target mRNA to be described (13–15). Since then, many other transcripts, including those coding for other pro-inflammatory cytokines, have been identified as TTP targets (12). Most attention has been focused on the primary role of TTP in myeloid cells, but cells that may play secondary roles in innate immunity, such as fibroblasts, also express TTP target transcripts, including those encoding chemokines, and this provides a mechanism for recruiting other leukocytes hypersecreting cytokines into the local site of inflammation (16, 17).

We recently developed a strain of knock-in mice in which we removed 136 b of the TTP mRNA 3’-untranslated region (UTR) (18). The result of this germ line manipulation was a decreased rate of decay of the ordinarily very labile TTP mRNA, resulting in increased levels of TTP mRNA and protein in tissues and cells. We hypothesized that these mice would be resistant to some models of inflammatory disease, and to date have demonstrated resistance to collagen-antibody induced arthritis, imiquimod-induced dermatitis, and experimental autoimmune encephalomyelitis (18, 19). More recently, these “TTPΔARE” mice have been shown to be resistant to a model of dental bone erosion and local inflammation (20).

Because of the resistance of the TTPΔARE mice to these models of immune/inflammatory disease, we hypothesized that they would be resistant to the development of EAU. We show here that homozygous TTPΔARE mice are indeed resistant to induction of EAU by a standard immunization procedure that normally provokes severe ocular inflammation in wild type (WT) control mice, whereas heterozygous animals were moderately susceptible. In addition, lymphocytes from immunized TTPΔARE mice were found to produce significantly lower amounts of pro-inflammatory cytokines than their WT controls, but higher levels of IL-10, an anti-inflammatory cytokine. TTPΔARE mice produced lower levels of serum antibodies to the immunizing antigen than their WT controls, but had more FoxP3^+^ T-regulatory (Treg) cells, which were moreover somewhat more efficient in suppressing effector T cells on a per-cell basis. Nonetheless, the TTPΔARE mice were capable of developing full-blown EAU in response to adoptively transferred activated WT T cells specific to the immunizing IRBP peptide, and their own T cells responded normally to TCR-driven stimulation *in vitro*. We propose that deficient antigen-presentation ability in TTPΔARE mice, together with increased Treg frequency and function, contribute to EAU protection.

## Materials and Methods

### Mice

TTPΔARE mice and their littermate WT controls, on a C57BL/6N background, have been described previously (18). Heterozygous mice were littermates from the same breeding colonies. All experiments were conducted on mice 8–12 weeks of age. Both male and female mice were used, with the sex being matched in all experiments. OTII mice were bred at NIH. The mice were housed in a specific pathogen-free facility, and all procedures were carried out in compliance with the NIH Resolution on the Use of Animals in Research, approved by the Animal Care and Use Committees of the National Institute of Environmental Health Sciences and the National Eye Institute.

### Induction of EAU and Disease Monitoring

Mice were immunized by subcutaneous injection of 0.2 ml of emulsion containing 150 µg of bovine interphotoreceptor retinoid-binding protein (IRBP) and 300 µg human IRBP uveitogenic peptide 1–20, in complete Freund’s adjuvant, as described in detail elsewhere (21). Pertussis toxin, 1 µg (Sigma, St Louis, MO), was injected intraperitoneally, concurrently with the immunization. The development of EAU was routinely monitored by fundoscopy, on days 9, 11, and 13 post-immunization (p.i.) to determine disease onset and progression. Mice were anesthetized with ketamine and xylazine (7:3) and retinas were examined under a binocular microscope after pupil dilation with 1% tropicamide (Bausch & Lomb) and 2.5% phenylephrine hydrochloride (Akorn). Disease Scores ranging between 0 to 4 were assigned to each eye using the inflammation scale described elsewhere (21). To take fundus images, mice were anesthetized with ketamine and xylazine (10:3) and the images were taken using a Micron III imager after pupil dilation with tropicamide and phenylephrine. For histological analysis, mice were euthanized on day 14 p.i. and eyes were collected, pre-fixed in 4% glutaraldehyde for 1 h and post-fixed in 10% buffered formalin. Fixed and dehydrated tissue was embedded in methacrylate or paraffin. Sections were cut through the pupillary-optic nerve plane and stained with hematoxylin and eosin. Severity of pathological changes was determined by using an inflammation and neurodegeneration scale of 0–4 in half point increments, as detailed elsewhere (22).

### Adoptive Transfer of EAU

EAU was adoptively transferred to naïve TTPΔARE mice and their WT controls by intraperitoneal injection of cells specific to peptide 651–670 of human IRBP (23). The pathogenic cells were generated as follows: spleen and draining lymph node cells of WT mice immunized with the peptide (150 µg/mouse) and pertussis toxin (1 µg/mouse) were collected on day 14 p.i. and cultured with the peptide for 48 h. Recipient mice were injected with 50 million cells/mouse and their eyes, collected 9 days later, were analyzed for histopathological changes.

### Analysis of Cellular Immune Responses

Mouse spleen and lymph node cells were collected after euthanasia, whereas peripheral blood lymphocytes were collected from mouse tails. The cells were cultured with different concentrations of IRBP, as detailed elsewhere (22, 24). Cytokine production was measured in culture supernatants collected after 48 h of incubation by the following ELISA kits: mouse IFN-γ was measured by ELISA Max™ Deluxe Set (Cat. Nr 430804) and IL-17A was measured by ELISA Max™ Deluxe Set (Cat. Nr 432504) from Biolegend (San Diego, CA). The following ELISA kits were purchased from R&D Systems (Minneapolis, MN): TNFα was measured by DuoSet ELISA kit (Cat. Nr DY410-05), IL-6 by DuoSet ELISA kit (Cat. Nr DY406-05), and mouse IL-10 by Quantikine ELISA kit (Cat. Nr M1000B).

### Quantitation of Treg Cells Expressing FoxP3

Cells expressing FoxP3 were identified among spleen and lymph node cells, as well as cells from peripheral blood, using the FoxP3 staining kit provided by eBioscience and following the manufacturer’s protocol. Cells were incubated with Fc block (clone 2.4G2, BioXcell) and stained with the following antibodies: eBioscience anti-CD45-FITC (30-f11), anti-CD4-PerCP ef710 (RM4.5) and FoxP3-PE (FJK-16s) were purchased from Invitrogen (Carlsbad, CA). Anti-CD25-APC (PC61.5) and anti-CD8-PE-Cy7 were purchased from Biolegend (San Diego, CA). For FoxP3 staining, eBioscience FoxP3/transcription factor staining buffer set (Cat. Nr 00-5523-00) (Invitrogen) was used according to the manufacturer’s protocol. Tonbo Biosciences Ghost Dye™ Red 780 (Cat No. 13-0865) was used for excluding the dead cells. The staining patterns were processed by CytoFLEX (Beckman Coulter, Brea, CA) and data were analyzed by FlowJo v. 10 (Tree Star, Ashland, OR, USA).

### Determination of Immunosuppressive Activity of Treg Cells

Treg suppression assays were carried out as described (25). Briefly, the assay was carried out in 96 well plates, with three purified/sorted cell populations in complete RPMI 1640 medium with 10% FBS: (i) irradiated (30Gy) T-cell-depleted splenocytes from WT mice (EasySep Mouse CD 90.2 positive selection kit II) (Stem cell technologies); (ii) CD4^+^CD25^-^ from WT mice; and (iii) CD4^+^CD25^HIGH^ cells from the TTPΔARE, or WT control mice, were sorted by FACS ARIAIII/Fusion cell sorter at the NEI flow cytometry core. Cell populations (i) and (ii) were added at 5 x 10^5^ in 50 μl/well, whereas cell populations (iii), from either WT or the TTPΔARE mice, were added in a series of double dilutions, in a volume of 50 μl/well. The T-cell populations were stimulated with 50 μl of anti-CD3 antibody (BioXcell) and incubated for 72 h, with 1 μCi of ^3^H thymidine being added for the last 8 h.

### Stimulation of Naïve T-Cells by Anti-CD3/CD28 Antibodies

T-cells were purified from naïve splenocytes of TTPΔARE and WT control mice using T cell enrichment columns (R&D systems). Enriched T-cells were stimulated *in vitro* using Dynabeads^®^ Mouse T-Activator CD3/CD28 (Thermo Fisher Scientific). Briefly, 1 x10^5^ cells per well, in 96-well plates, were stimulated with different volumes of pre-washed CD3/28 beads for 48 h, following the manufacturer’s suggestions. Stimulation levels were measured by a conventional ^3^H thymidine incorporation assay for lymphocyte proliferation and culture supernatants were collected for testing cytokine production.

### Antigen Presenting Cell (APC) Assay

Irradiated (30 Gy) splenocytes from TTPΔARE mice or WT controls were plated at 2 X 10^5^/well in 96 well plates. Naïve T cells from OTII mice were purified using naïve T cells isolation kit (Stem Cell technologies) and plated at 1 X 10^5^/well. OVA peptide 323–339 was used at three different concentrations, 10 mg/ml, 1 mg/ml, and 0.1 mg/ml, respectively. The plates were pulsed with ^3^H thymidine after 48 h of incubation and harvested 16 h later.

### Serum Antibodies

Mouse sera were collected upon euthanasia, and the levels of serum antibodies against IRBP were measured by conventional ELISA (7). The antibody isotypes to be examined included total IgG, IgG1, and IgG2a.

### Statistical Analysis

GraphPad Prism 8.0 was used for statistical analysis. Mann-Whitney U test (non-parametric data) or student’s t-test (parametric data) was used for two-group comparisons. A p-value of <0.05 was considered statistically significant. Data are displayed as mean ± SEM. Two-way ANOVA with Tukey’s *post hoc* test per factor was used for comparison of two factors of interest among three groups.

## Results

### TTPΔARE Mice Were Protected From EAU Induction

**Figure 1:**
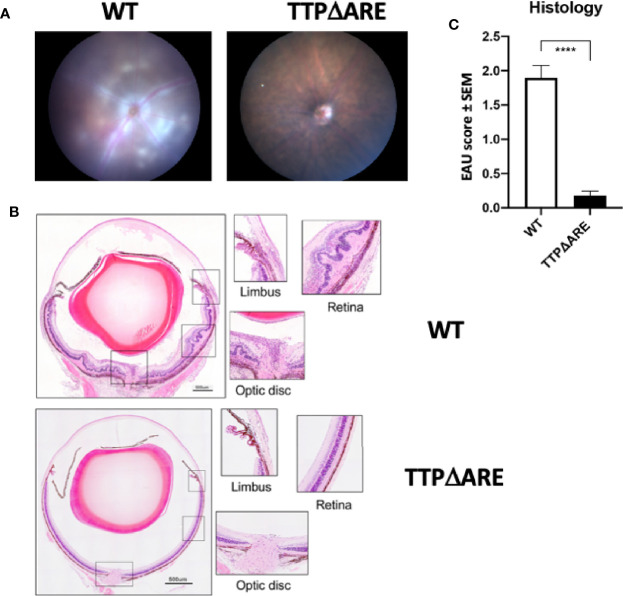
Homozygous TTPΔARE mice are resistant to experimental autoimmune uveitis (EAU) development. Shown are **(A)** fundoscopic images (day 13 p.i.) and **(B)** histological images (day 14 p.i.) of eyes from homozygous TTPΔARE mice and their WT controls. The fundoscopic changes in the TTPΔARE mouse include vascular leakage due to inflammation, while the histological changes include retinal folding with accumulation of serous and cellular exudates in the subretinal space. The typical histological EAU changes also include inflammatory cell infiltration throughout the retinal layers, as well as accumulation of cells in the vitreous, optic nerve head and limbus. **(C)** summarizes the quantitative histological data from four separate experiments, with a total of 22 mice in each group. Mann-Whitney test was used, ****p<0.0001.

To test the susceptibility of TTPΔARE mice to EAU induction, we immunized groups of these mice with IRBP and p1-20 as detailed above. Littermate WT mice, of the same age and sex were used as controls. Disease severity was assessed by fundoscopy and fundus images. Representative fundus images from WT and TTPΔARE mice are shown in **Figure 1A**. Tissue damage analyzed by histopathology is shown in **Figure 1B**. Moderate to severe disease changes were observed in all WT mice, whereas very little or no disease was found in the homozygous TTPΔARE mice. Histopathological analyses were carried out on tissues from four separate experiments, with a total of 22 mice in each group. The data are summarized in **Figure 1C** and demonstrate highly significant differences in inflammation and tissue damage between the two mouse genotypes.

### TTPΔARE Mice Have Increased Frequency and Function of FoxP3^+^ Treg Cells Compared to WT Controls

**Figure 2:**
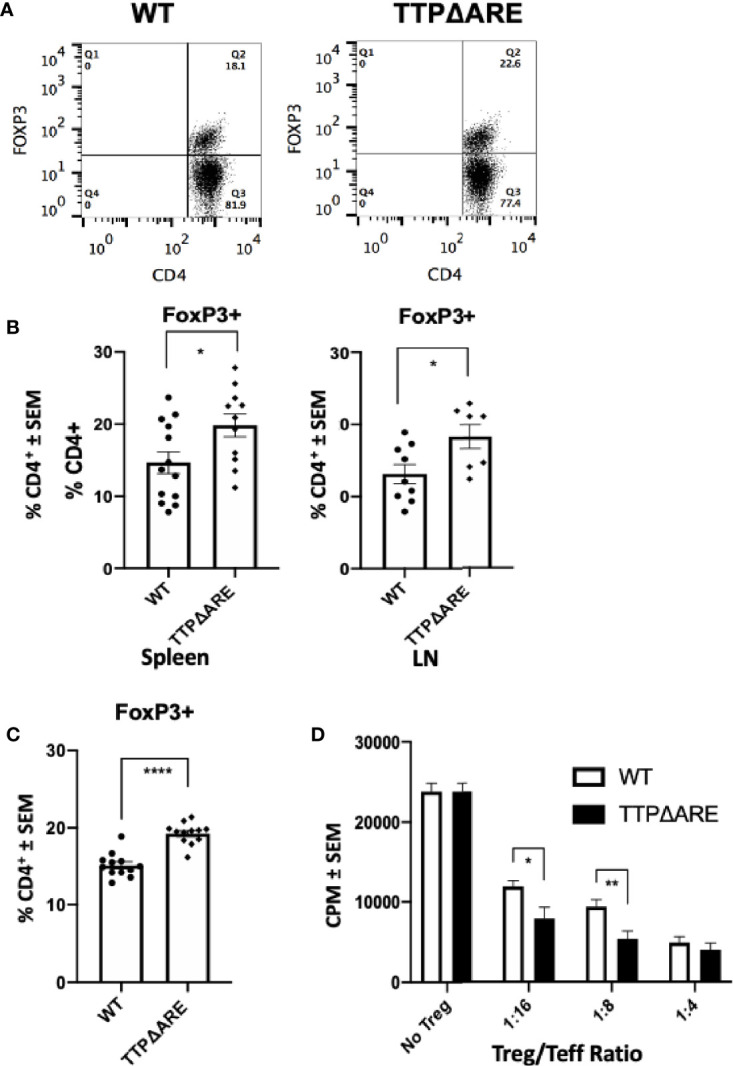
The proportion of FoxP3+ regulatory cells and their inhibitory function are higher in TTPΔARE mice than in their WT controls. **(A)** Shows flow cytometric data from a typical experiment, demonstrating the higher proportion of FoxP3+ cells among spleen cells from the TTPΔARE mice than from the WT controls, gated on CD4^+^ population. **(B)** Summarizes three combined experiments with spleen and two experiments with LN cells. The spleen cells were collected from 13 WT and 11 TTPΔARE mice, whereas the LN cells were collected from nine and seven mice, respectively. Each dot represents data from an individual mouse. **(C)** Shows the relative proportions of FoxP3+ cells among peripheral blood cells from TTPΔARE mice and their WT controls. The data are values from 10 mice in each group. **(D)** The regulatory function of Treg cells from the two mouse genotypes was determined by the level of their suppression of the proliferative response of naïve T-cells stimulated by anti-CD3 antibody (see *Materials and Methods* for details). The figure combines data from two experiments. t-test was used for statistical comparisons, *p < 0.05, **p < 0.01 and ****p < 0.0001.

To determine the possible involvement of T regulatory (Treg) cells in the apparent EAU resistance of the TTPΔARE mice, we measured their proportions in spleen and draining lymph node cells from immunized TTPΔARE mice and their WT controls, using flow cytometry. **Figure 2A** shows flow cytrometric data from a typical experiment, whereas **Figure 2B** summarizes three combined experiments with spleen (total 13 WT and 11 TTPΔARE mice) and two combined experiments with LN cells (total nine and seven mice, respectively). The data show statistically higher frequencies of FoxP3+ cells in TTPΔARE mice than in controls. In addition, we examined the levels of FoxP3+ cells in non-immunized mice, using peripheral blood CD4^+^ cells. The data collected with peripheral blood samples from 10 individual mice from each mouse genotype are shown in **Figure 2C**. The proportions of FoxP3+ cells were significantly higher in CD4^+^ cells from TTPΔARE mice than WT controls at steady state.

To further elucidate the suppressive function of Treg cells in TTPΔARE mice, we conducted a standard Treg suppression assay. Sorted populations of CD4+CD25^HIGH^ cells from TTPΔARE mice were compared with those of the WT controls for their ability to inhibit anti-CD3 stimulated CD4^+^CD25^-^ effector T-cell proliferative response in the presence of irradiated WT splenocytes as APC. The data shown in **Figure 2D** indicate that the Treg cells of the TTPΔARE mice were demonstrably more suppressive than their WT controls on a per-cell basis.

### Heterozygous Mice Develop EAU and Related Immune Responses Intermediate Between Homozygous TTPΔARE Mice and WT Controls

**Figure 3:**
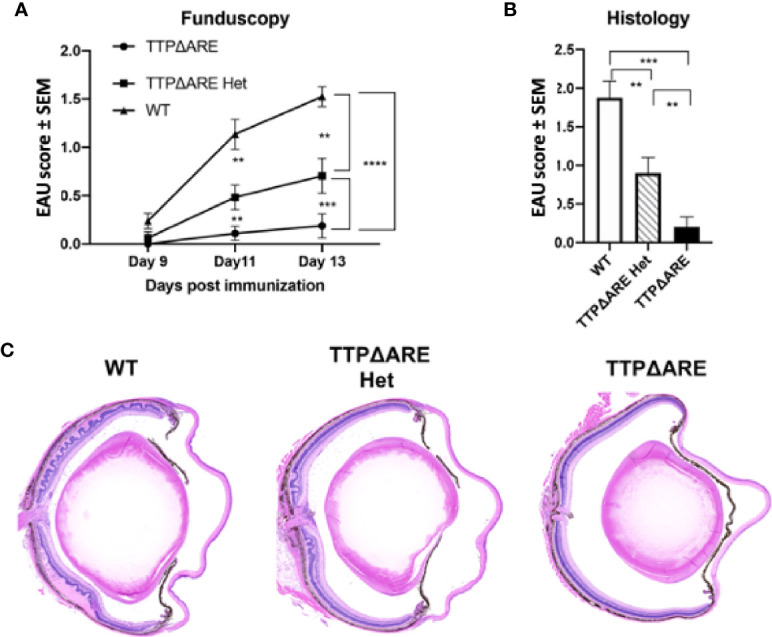
Heterozygous mice develop experimental autoimmune uveitis (EAU) of intermediate severity. Groups of mice from the three genotypes were immunized for EAU induction, and the development of disease was measured by fundoscopy **(A)** or histology **(B)**. The figure summarizes data from two separate experiments, with five mice in each group. **(C)** Representative histological sections of eyes of the three mouse genotypes. Mann-Whitney U test was used for statistical comparisons, **p < 0.01, ***p < 0.001, ****p < 0.0001.

**Figure 4:**
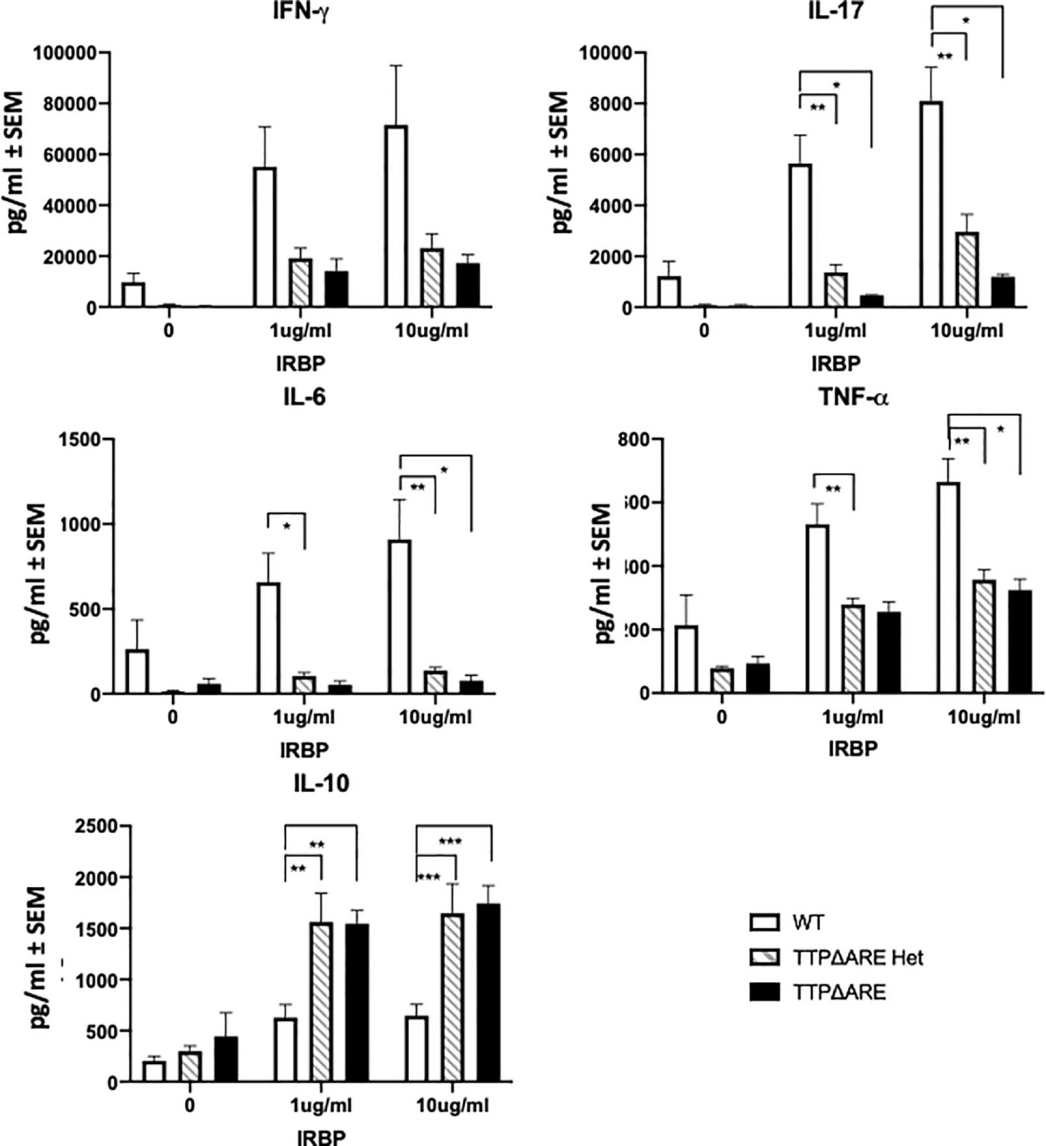
Cytokine production by lymphocytes from TTPΔARE homozygous and heterozygous mice and their WT controls. Lymph node cells from the immunized mice of the three genotypes were incubated with IRBP at 1 or 10 µg/ml, and their supernatants were collected after 2 days (or 3 days for IL-10), and tested for the indicated cytokines, t-test was used for statistical comparisons: *p < 0.05; **p < 0.01; ***p < 0.001.

**Figure 5:**
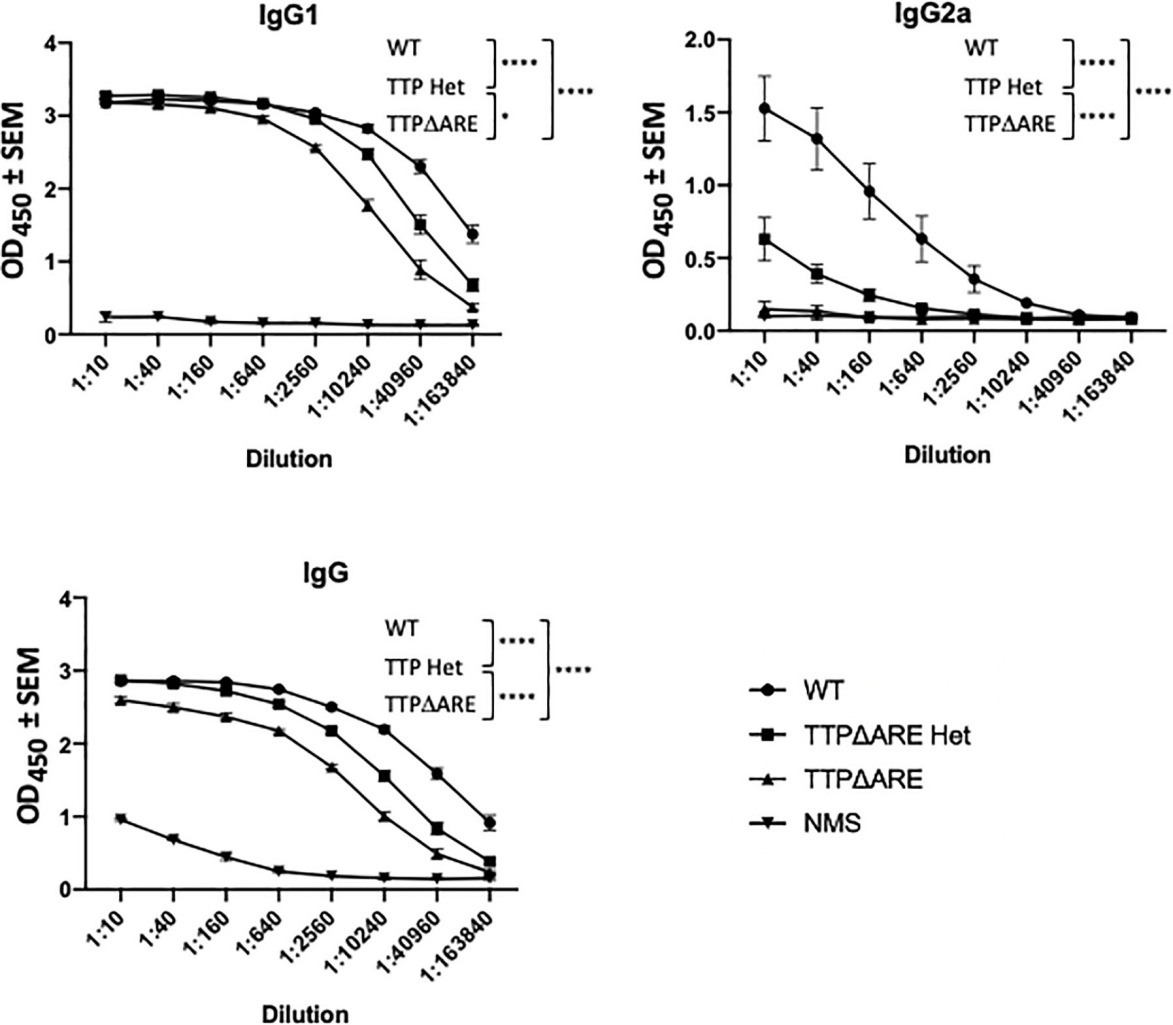
Antibody production by cells of TTPΔARE homozygous and heterozygous mice and their WT controls. Sera from mice of the three groups were analyzed for anti-interphotoreceptor retinoid-binding protein (IRBP) antibodies by ELISA. The antibody isotypes were determined using the appropriate antibodies for hybridization. Shown are the combined data from eight individual homozygous TTPΔARE mice, 10 heterozygous mice, and 10 WT controls. Sera were collected from the same mice whose cytokine responses are quantitated in **Figure 4**. Two-way ANOVA with Tukey’s *post hoc* test was performed for statistical comparisons between groups. *p < 0.05; ****p < 0.0001. NMS, normal mouse serum, collected from naïve C57BL/6 mice, was used as negative control.

To determine whether the protective effect of increased TTP against induction of EAU was related to gene dose, we compared the development of disease and related immune responses of the heterozygous mice with those of the of the WT and homozygous TTPΔARE mice. These data are summarized in **Figures 3**–**5**, with data collected from two separate experiments yielding similar results. In most cases, the responses of the heterozygous mice were intermediate in severity between the responses of the homozygous TTPΔARE mice and the WT control mice, as follows:

*EAU severity*. **Figure 3** demonstrates that EAU, as measured on day 14 by both fundoscopy (A) and histology (B), was intermediate in severity between the TTPΔARE mice and their WT controls. The mild, or no, disease that we observed in the TTPΔARE mice did not progress to become more severe at later time points.

*Cytokine production*. **Figure 4** shows the levels of five major cytokines in supernatants of draining lymph node cells from the three experimental groups, cultured with IRBP at 1 or 10 µg/ml. IFN-γ, IL-17, IL-6, and TNFα are pro-inflammatory, whereas IL-10 is an anti-inflammatory cytokine (8, 26). The levels of the pro-inflammatory cytokines secreted by cells from WT mice were generally significantly higher than those from the TTPΔARE homozygous or heterozygous cells. In contrast to the four pro-inflammatory cytokines, the production of IL-10, the anti-inflammatory cytokine, was lower in cultures of the WT cells than in cultures from the homozygous or heterozygous TTPΔARE mice.

*Antibody production*. **Figure 5** shows IRBP specific antibody levels in individual sera from each mouse group, as measured by ELISA. The levels of total IgG and IgG1 antibodies in sera from WT mice were significantly higher than the levels in sera from homozygous mice, whereas the antibody levels from heterozygous mice were intermediate between those of the two other mouse genotypes. Interestingly, the differences among the three mouse genotypes were particularly evident in their production of IgG2a antibody: essentially no IgG2a antibody to IRBP was detected in sera from the homozygous TTPΔARE mice, intermediate levels of this antibody isotype were found in sera from the heterozygous mice, and high levels of IgG2a antibody were produced by the WT mice.

### EAU Can Be Induced in TTPΔARE Mice Using Adoptive Transfer of IRBP-Specific T-Cells

**Figure 6:**
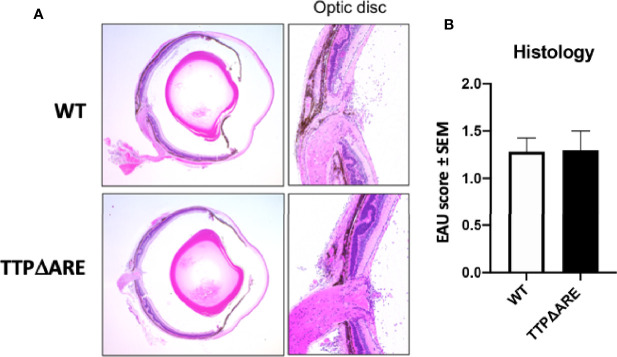
TTPΔARE mice resemble their WT controls in development of experimental autoimmune uveitis (EAU) by adoptive transfer. Naive mice from the two genotypes were injected with 50 million T-cells specific to the IRBP peptide 651-670, from immunized C57BL/6N donor mice, and the presence and severity of EAU were determined by histological analysis of eyes after euthanasia on day 9 post cell injection. **(A)** shows that the eyes from both genotypes of mice exhibited similar pathological changes, including mild retinal folding and infiltration of inflammatory cells into the optic nerve head, retinal tissues and vitreous. **(B)** shows the pathological changes. The Mann-Whitney U test was used for statistical comparison. Combined data from two experiments.

In order to test whether the histopathological changes typical of EAU can develop in the eyes of the TTPΔARE mice under different circumstances, we used an adoptive transfer system in which the disease is transferred and mediated by activated lymphocytes specific to IRBP or its pathogenic peptide (amino acids 651–670) (23). EAU from adoptive transfer developed considerably earlier than the conventional disease. Pathological changes were detected by fundoscopy in recipients of IRBP specific T-cells beginning 5 days post-transfer (**Figure 6**), as compared with day 9 in actively immunized mice (**Figure 3**). Importantly, the levels of disease severity, as detected and measured by either fundoscopy or histology, were similar in the recipient TTPΔARE mice and their WT controls (**Figure 6**). These results demonstrate that the homozygous TTPΔARE mice can develop EAU similarly to their WT controls when disease is induced by lymphocytes sensitized to the uveitogenic antigen, indicating that in this system the contribution of host-derived cytokines does not affect disease progression.

### T Lymphocytes From TTPΔARE Mice Resemble Those of WT Controls in Their Proliferative Response to Anti-CD3/CD28 Antibodies

**Figure 7:**
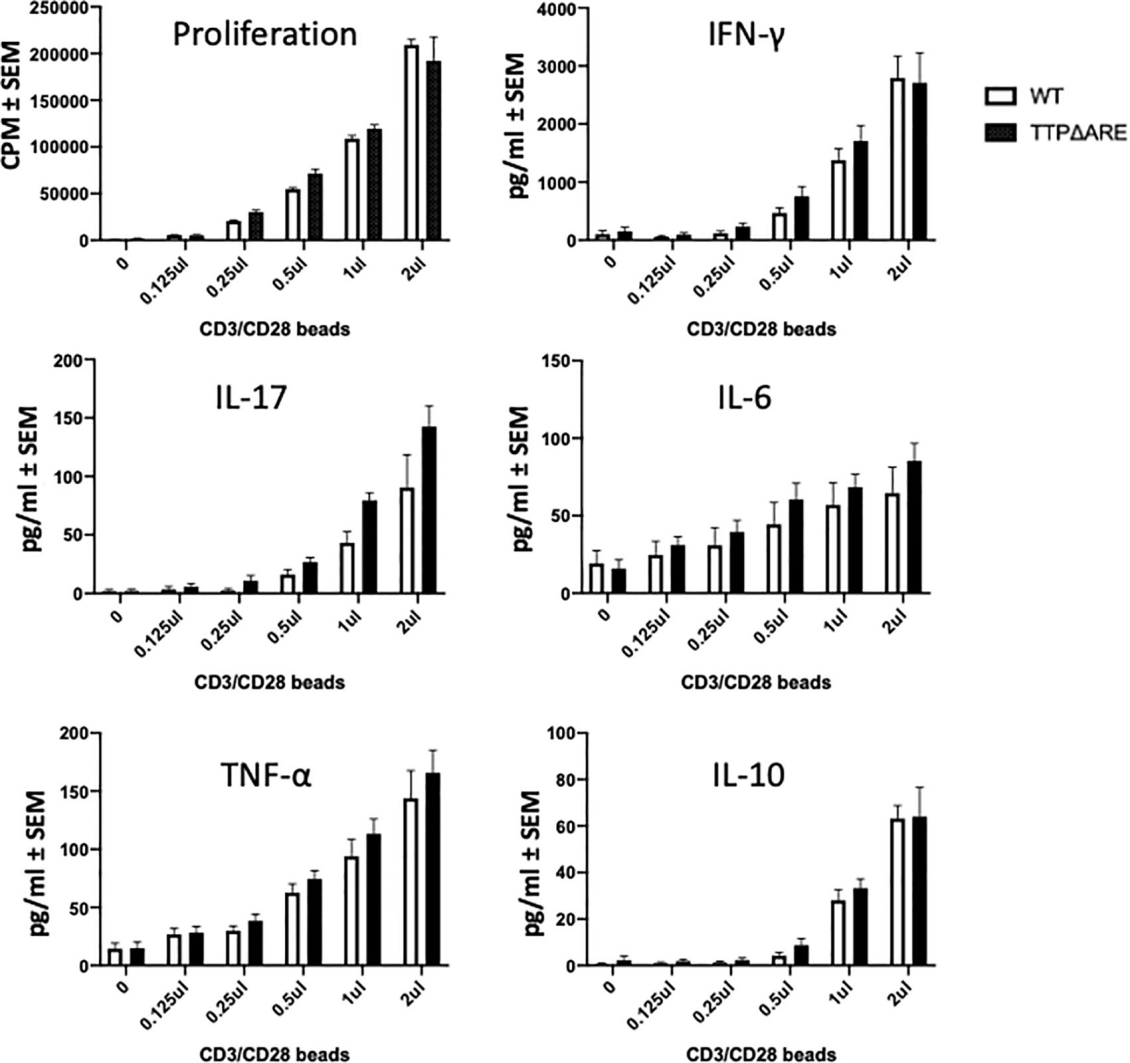
T-cells from TTPΔARE mice resemble their WT controls in their response to stimulation by beads coated with anti CD3/CD28 antibodies. CD3 column-enriched T cells were stimulated with increasing volumes of the beads for 48 h. Lymphocyte stimulation was determined by proliferation assay and by release of cytokines. t-test was used for statistical comparisons. Combined data of three experiments.

Resistance to the development of a pathogenic immune response such as EAU could be attributed to a defect in the ability of T-cells to respond to specific antigenic stimuli presented on antigen-presenting cells (APC). The complex multi-molecular process of antigenic stimulation can be mimicked by incubating naïve T-cells with antibodies against CD3 and CD28 molecules for T cell receptor signaling (27). We used this approach to examine whether T-cells from homozygous TTPΔARE mice were capable of being stimulated by this procedure. The response of TTPΔARE cells was compared to that of WT mouse cells and the data from three experiments are shown in **Figure 7**. No differences were noted between the responses of the cells from the two genotypes by proliferation or cytokine release (**Figure 7**), indicating that the inability of the TTPΔARE mice to develop typical EAU is not due to inability of their T-cells to respond *via* T cell receptor activation.

### TTPΔARE APC Are Deficient in Their Antigen Presentation Ability

**Figure 8:**
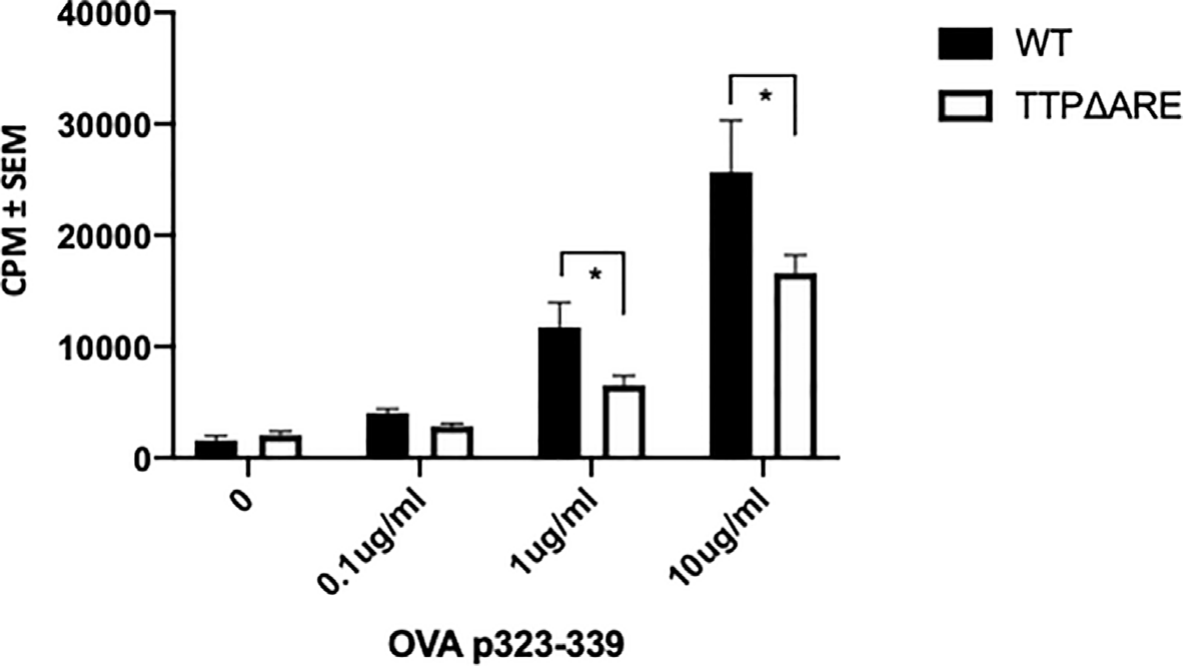
TTPΔARE mice are deficient in their antigen presentation ability. Naïve CD4^+^ T cells from OTII mice were cultured with irradiated splenocytes from TTPΔARE mice, or WT controls, in the presence of OVA323-339 peptide for 48 h. The responses were determined by the incorporation of tritiated thymidine. t-test was used for statistical comparisons, *p < 0.05. Combined data of two experiments.

Antigen presentation by APC to T-lymphocytes can determine the magnitude of the immune response. We, therefore, examined the ability of APC from TTPΔARE mice to prime naïve T cells. We used naïve CD4+ T cells from the OVA-TCR transgenic OTII mice. As demonstrated in **Figure 8**, APC from TTPΔARE mice were significantly less efficient than their WT controls in presenting the cognate antigen (OVA323-339) to OTII T-cells. This finding suggests that deficiency in antigen presentation plays a crucial role in the reduced immune response of TTPΔARE mice.

## Discussion

The data reported here demonstrate that the TTPΔARE mice are protected against the development of EAU, a well-defined animal model for non-infectious human uveitis. There was evidence of a gene dose effect, with homozygous TTPΔARE mice being essentially resistant to EAU, whereas heterozygous mice developed disease at levels intermediate between homozygous mice and their WT controls. We then performed experiments to examine potential mechanisms of this protective effect.

The pathogenic process of EAU is generally thought to be initiated by cytokines released by Th1 and Th17 lymphocytes sensitized against the retinal antigen upon their exposure to the target retinal molecule. We therefore analyzed the major cytokines released in lymphocyte cultures incubated with IRBP. Lymphocytes from the homozygous and heterozygous mice differed from their WT controls by lower levels of production of the pro-inflammatory cytokines, IFN-γ, IL-17, TNFα, and IL-6, several of which are known targets of TTP. In contrast, their production of IL-10, an anti-inflammatory cytokine, was higher than that of the WT controls. Remarkably, cells of the heterozygous mice produced these cytokines at levels roughly intermediate between the WT and TTPΔARE cells, again indicating a gene dose effect of TTP on these biological processes.

Antibodies are produced by B-cells, but the “help” of T-cells is crucial for the process. In line with the T-cell response data, the TTPΔARE mice essentially failed to produce IgG2a antibodies, whereas the levels of this antibody isotype were again intermediate in the heterozygous animals between those of the homozygous and WT controls. IgG2a is used as a marker for Th1 responder cells (28, 29). It is therefore of great interest that the homozygous TTPΔARE mice essentially failed to make detectable IgG2a antibodies, suggesting that production of these antibodies is highly susceptible to increased levels of TTP. It will be interesting to determine whether these antibodies are overproduced in conventional or conditional TTP KO mouse models.

Another mechanistic possibility was suggested by a recent study in which TTP was found to be induced by iron deficiency (30). The increased TTP was thought to decrease the expression of mRNAs encoding the mitochondrial Fe/S cluster-containing proteins NDUFS1 and UQCRFS1. Conversely, in TTP KO mouse hearts, UQCRFS1 levels were not decreased in iron deficiency, resulting in abnormalities of Fe/S cluster function, oxidative damage, and cardiac dysfunction in the setting of iron deficiency. This study suggests the intriguing possibility that overexpression of TTP by other means, as in the present paper, might improve mitochondrial health by regulating levels of Fe/S cluster-containing proteins through their encoding mRNAs.

The dampened effector T (and consequently B?) cell responses secondary to enhanced levels of TTP could be caused either by tissue-specific, or T cell-intrinsic, or an APC-driven defect. Adoptive transfer of pathogenic WT T-cells induced similar pathological changes in eyes of TTPΔARE mice and their WT controls, thus ruling out potential deficiencies in ocular cells and tissues of the mutant mice in the pathogenic process of EAU induction, including host-derived cytokines that would be regulated by TTP in the process of tissue damage. Effector cytokines produced by the donor cells were necessary and sufficient to induce pathology. We ruled out an intrinsic defect in the response of TTPΔARE T cells by demonstrating that their proliferative response to stimuli by anti-CD3/CD28 beads, which circumvent the need for antigen and APC, was reminiscent of WT controls (**Figure 7**). Similar responses were also seen to the T cell mitogen PHA (data not shown). These observations indicate that T-cells from TTPΔARE mice respond normally to TCR-driven stimulation. Rather, the reduced ability of TTPΔARE APC to prime naïve OT-II OVA-specific T cells indicates that the dampened immune response appears to be largely secondary to the reduced ability of TTPΔARE APC to present antigen.

Many immunopathogenic processes are physiologically inhibited by the family of FoxP3+ Treg cells (31, 32). We found that the frequency of FoxP3+ cells in the TTPΔARE mice was significantly higher than in their WT controls. Further, Treg cells from the TTPΔARE mice had increased suppressive function on a per-cell basis compared to their WT controls. These findings suggest a likely involvement of Treg cells in the disease prevention seen in these mice. Since Tregs, like T effector cells, require APC for activation, it is unclear how the functionally deficient TTPΔARE APC might support an enhanced Treg function.

Other mechanisms may be involved in the observed Treg modulation. For example, previous work in mouse embryonic fibroblasts (MEF) established that overexpression of TTP led to decreased levels of transferrin receptor (Tfrc) mRNA, whereas levels of Tfrc mRNA were increased in TTP KO cells (33); the increase in the KO cells was accompanied by increased stability of the Tfrc mRNA. In WT MEF, rapamycin also induced TTP and decreased Trfc mRNA levels and stability, effects that were blunted in the TTP KO cells. The authors suggested that TTP was downstream of mTOR, and that TTP might be responsible for at least some of the effects of mTOR on Tfrc mRNA levels. The clinical relevance of these findings was supported by a recent study in which rapamycin treatment of patients with systemic lupus erythematosus (SLE) led to improvement in disease activity, while correcting abnormalities in proinflammatory T-cell lineage specification and reducing the production of proinflammatory cytokines (34). A separate study found that mTOR activity was increased in Treg cells from patients with SLE, and that treatment with rapamycin corrected the Treg dysfunction (35). These studies suggest a role for TTP in the regulation of Treg activity that involves the mTOR pathway.

Taken together, our results demonstrate that TTPΔARE mice are remarkably resistant to the development of EAU, a model of inflammatory eye diseases. The predicted mechanism is that the elevated levels of TTP in virtually all cells and tissues restrict the ability of the TTPΔARE mouse to mount a pathogenic cytokine response after immunization, presumably by destabilization of their mRNAs, and the data shown here support that suggested mechanism. EAU thus joins other mouse models of immune and inflammatory diseases to which the TTPΔARE mice were resistant, including collagen antibody-induced arthritis, imiquimod-induced dermatitis, and experimental autoimmune encephalomyelitis (18), and a bacterial model of dental inflammation (20). Notably, our data shed new light on the mechanisms of the protective effect of enhanced TTP expression.

These data suggest the possibility that increasing systemic or even possibly local levels of TTP might be considered as possible therapeutic strategies in diseases such as non-infectious uveitis. We have suggested various approaches to TTP-based therapies in a recent review (19). That said, animals with a reduced effector response and an enhanced expression of regulatory response could also be more resistant to immunization in general, including reduction of beneficial host immunity, as is seen, e.g., with systemic TNFα neutralization. This could have implications for systemic use of TTP manipulation as a therapeutic approach. However, the eye is unusual if not unique in opening up possibilities for local therapy, perhaps using locally instilled small molecule drugs or anti-sense oligonucleotides. Other methods are being developed to provide local gene therapy to the eye by expressing anti-inflammatory proteins from viral vectors (36, 37), or in direct plasmid transfection (38). Experiments with this type of local gene therapy would also be useful in determining the extent to which local elevations of TTP, as opposed to the systemic elevations seen in this model, might be beneficial in controlling the local inflammation. If successful, local targeting of TTP might also address the concern that systemic targeting of TTP, with consequent reduction in effector cytokines and enhancement of immunosuppressive responses, would adversely affect host immunity to pathogens.

In summary, our data show for the first time the remarkable ability of elevated TTP levels to protect against the development of ocular autoimmunity. These observations also support the potential use of this experimental system in the development of new treatments for uveitis and similar diseases with elements of autoimmune pathogenesis.

## Data Availability Statement

The raw data supporting the conclusions of this article will be made available by the authors, without undue reservation.

## Ethics Statement

The animal study was reviewed and approved by NEI ACUC.

## Author Contributions

BX: conducted EAU experiments, ELISA, FACS data analysis, and manuscript. JT: conducted experiments, FACS data analysis, and manuscript preparation. CL: conducted experiments, ELISA, and data analysis. WW: conducted EAU experiments and FACS data analysis. DS: bred TTP mice. MM: conducted adoptive transfer experiments. RH: conducted experiments, interpreted data. RC: designed experiments and manuscript preparation. PB: designed experiments and manuscript preparation. IG: conceptualized and supervised study, designed experiments, wrote manuscript, and finalized manuscript. All authors contributed to the article and approved the submitted version.

## Funding

US Government funding through NEI and NIEHS. Supported by the Intramural Research Programs of the NIH, NIEHS (DS and PB), and NEI (BX, JT, CL, WW, MM, RH IG, RC).

## Conflict of Interest

The authors declare that the research was conducted in the absence of any commercial or financial relationships that could be construed as a potential conflict of interest.
